# Ancillary and instrumental body movements during inhalation in clarinetists

**DOI:** 10.3389/fpsyg.2024.1394035

**Published:** 2024-05-22

**Authors:** Manfred Nusseck, Anna Immerz, Jesper Hohagen, Claudia Spahn

**Affiliations:** Freiburg Center for Research and Teaching in Music, Freiburg Institute for Musicians’ Medicine, University of Music Freiburg, Medical Faculty of the Albert-Ludwigs-University Freiburg, Freiburg, Germany

**Keywords:** ancillary movements, instrumental movements, inhalation process, movement behavior, body posture

## Abstract

**Background:**

Playing a musical instrument requires physical movements that are involved in sound production and movements with more expressive and communicative characteristics. Both movements co-occur during a performance; however, the interaction between the movements is still unclear.

**Methods:**

Using motion capture technology, the movement patterns of clarinetists were analyzed at certain points in a performance to investigate how instrumental and ancillary movements interplay. Movements in the arms and knees of clarinetists during a performance were recorded using this technology. The mean angular movements at specific points in the piece, where some players inhaled and others did not, were compared.

**Results:**

While the players who inhaled adopted significantly more upright body and neutral arm positions, the players who did not inhale seemed less interrupted in their performance. The results showed that the players performed rather individual ancillary movements, but at specific points, such as during melodic transitions, they performed similarly. At certain points in the melody, while some players needed to inhale, others adjusted their playing according to the inhalation moment to adopt a suitable body position.

**Discussion:**

The ancillary movement was consequently interrupted by the physiological necessity to inhale. The findings provide more insights into the interplay of instrumental and ancillary movements during a performance.

## 1 Introduction

For musicians, body movements are essential to perform musical content and create an expressive moment (Leman, 2016). Musicians’ body movements can be categorized into different functions associated with auditory and visual aspects of the performance (Godøy and Leman, 2010). First, *instrumental movements* are necessary because they are required for sound production. These movements are restricted by the instrument and the notes to be played. They occur in rather similar ways in repeated performances.

In addition to the instrumental movements, musicians perform body movements that are not essential for the production of sound, but follow more intentional, communicative, and expressive purposes, the so-called *ancillary movements* (Jensenius et al., 2010; Nusseck et al., 2018). These movements contain individual aspects and convey the personal characteristics of the musician (Godøy and Leman, 2010). Musicians use both instrumental and ancillary movements simultaneously while playing (Dahl et al., 2010).

*Ancillary movements* can appear in head movements, facial expressions, and side-to-side swaying (Davidson, 2012). For instance, body sways can transport emotional expressions (Demos et al., 2018) and create a shared emotional expression in a musical ensemble (Chang et al., 2019). These movements reflect musical structures, such as melodic changes and transitions (MacRitchie et al., 2013), as well as dynamics (Nusseck et al., 2022), and can aid tempo perception (Massie-Laberge et al., 2019). When studying the movements of professional pianists, it was found that certain movement strategies were used not only to express musical goals but also to compensate for individual physical constraints in order to economize movements (Turner et al., 2021).

Ancillary movements are highly individual in style and execution. However, at certain musical positions, similar movement gestures have been identified across players. Similar movements of pianists have been observed at structurally prominent positions in the score (Thompson and Luck, 2012). For clarinetists, similar movements of the bell were found across players at specific points, such as a melodic or rhythmic transition and the ending (Teixeira et al., 2015). Congruent movements were also related to key musical moments and specific, musically expressive contents. In saxophone players, knee flexions were identified as ancillary movements with an expressive purpose and were found to be performed in anticipation of specific melodic phrases (Moura et al., 2023). The knee movements were associated with pitch expectations and rhythmic density, suggesting that they are related to expressive and facilitative qualities.

In clarinet playing, ancillary movements have been largely researched (Nusseck et al., 2018). It was found that these movements mainly involve bending the knees and raising the arms (Weiss et al., 2018). At certain musical transitions or endings, ancillary movements across players become very similar, and the players adopt a rather neutral and upright body posture (Nusseck et al., 2022).

For wind instruments, breathing plays a decisive role in sound production. During a performance, the moment of inhalation needs to be considered and anticipated. The inhalation itself can be seen as an instrumental movement to prepare for sound production and must be integrated into the context of the performance. In previous studies, clarinetists played a piece in which inhalation coincided with transitions or endings of melodic phrases (Nusseck et al., 2022). The players performed at those points with a more upright body posture and neutral arm positions. As these ancillary movements are also associated with certain expressive and communicative aspects, it is difficult to distinguish whether these movements also follow a biomechanical purpose, to position the body in a physiologically supportive posture for optimal inhalation. Experienced performers certainly combine expression with movement, but it is not clear how ancillary movements constitute and support the player’s technical execution (Moura et al., 2023).

This study aimed to investigate the body movements of clarinetists during the performance of a melody. Typically, ancillary movements are predominant in such performances, but there may also be a need for inhalations. By comparing the movements of players who inhaled at a particular point in the melody with players who did not inhale at that point, the results provide insights into the potential physiological functions related to inhalation. Therefore, a piece with a slow and long melody that required players to inhale while performing was selected. Movements in players during inhalation, at the end of a melodic phrase, and during the melody were observed and analyzed.

## 2 Materials and methods

### 2.1 Participants

The recordings of clarinet players used for this study are a subsample of the dataset reported by Weiss et al. (2018). Of the original 22 clarinet players, 20 players who had complete sets of movement and audio data were selected. The average age was 32.3 years (*SD* = 12.6 years). The players were at a professional instrumental level with a mean duration of playing the clarinet of 22.2 years (*SD* = 12.1 years).

**Figure 1:**
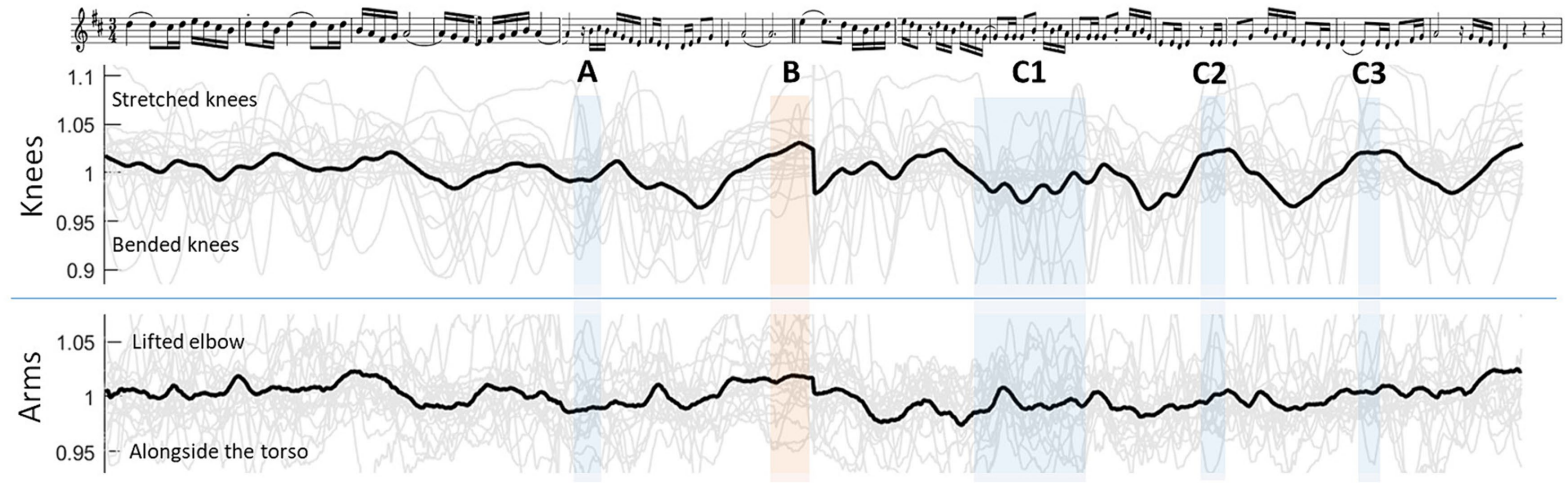
Top row: Score of Ravel’s “*Boléro*” theme created with LilyPond. Second and third rows: Trajectories of the angular movement in the knees and arms (Note: values were divided by the mean angle; therefore, there is no unit. One is identical with the mean angle. In grey: each player. In black: mean of all players). A-C and marked with light blue: specific points in the theme where players inhaled (in light orange: the middle of the theme). Please note that the score is slightly offset from the movement curves due to individual tempo variations and different lengths between written and played notes. Therefore, the score has been compressed or stretched at some points to adapt it to the timeline of the movement.

The clarinetists played the theme of Maurice Ravel’s “*Boléro*” one time. They were asked to practice the piece before the recording session and to play the piece as they would normally play it in front of an audience. The theme is a two-part theme with 16 bars that requires inhalation between and during the parts. The theme score is shown in the top row of Figure 1, which was created with LilyPond.^1^ A music sheet was placed on a music stand approximately 60 cm before the players. The tempo was provided with a metronome (77 bpm) before the performance. During the performance, the musicians did not play with the metronome. All players provided their written consent to participate in the study.

### 2.2 Measuring technics

For the motion analysis, the clarinetists were recorded using an optical 3D motion capture system. Their performances were captured by four calibrated digital videos with a sampling rate of 50 frames per second using Templo software (Contemplas GmbH, Kempten, Germany). In total, 22 markers were attached to central joints and body parts to obtain a whole-body image, and the marker positions were digitized using Peak Motus 10 (Vicon). In addition, the players were acoustically recorded using an audio recorder (Zoom H4N), which was placed approximately 2 m in front of the player. More details regarding the recording system can be found in the studies by Weiss et al. (2018) and Nusseck et al. (2022).

### 2.3 Analyses

Based on previous studies (Weiss et al., 2018; Nusseck et al., 2022), the angular movements of the knees and arms were considered for analysis. The knee angle was calculated between the foot and the hip marker, using the knee marker as the angle intersection point. A high value indicates an extended leg, while a low angle indicates a bent knee. The knee angle measurement cannot be greater than 180 degrees due to anatomical limitations. The lowest angle found was approximately 100 degrees. Both knee angles were highly correlated (*r* > 0.9). Therefore, only one knee angle (right knee) was used for the analysis.

The arm angle was calculated using the elbow, shoulder, and neck markers. A high value indicates an opening in the armpit. With a low value, the arms were held close to the trunk. The value ranged between 90 and 180 degrees. Both arm angles correlated highly with each other (r > 0.8), and thus, one arm angle was used for the analysis. As the right arm also has a holding function of the clarinet, the left arm movements were chosen.

Even with a given general tempo, the players performed rather individually. Therefore, the recordings were resampled to an equal length. This resampling was conducted for both parts of the theme separately and was then put together.

The measured knee and arm values of each player were divided by the mean of their movement trajectory to normalize individual angle differences. The resulting value is therefore scaled approximately 1 and has no unit. For instance, a value of 1.02 represents an angular difference of approximately 3–5 degrees higher than the mean angle.

The spectral centroid (SC) was used in the audio data to identify the positions when the players inhaled. The SC indicates where the center of mass of the frequency spectrum lies. Inhaling produces a type of noise in which the frequency spectrum is difficult to recognize, and the SC is very high compared to playing a sound. Using the Matlab MIRToolbox (Lartillot et al., 2008), the points at which the players inhaled were identified and used for calculating the angular movements of the knees and arms at these positions.

### 2.4 Statistics

The statistical analyses were performed with SPSS (version 28, Armonk, NY: IBM Corp.). Descriptive statistics were calculated for parametric variables, including mean values and standard deviations (SD). For parametric comparisons, analyses of variances (ANOVAs) were used. Simple comparisons of mean values to a single value were performed with two-sided *t*-tests. The level of statistical significance was set at *p* = 0.05.

## 3 Results

On average, the players inhaled 3.1 times (SD = 1.0) during the theme. The number was distributed rather evenly between two (*n* = 7 players), three (*n* = 6 players), and four (*n* = 7 players) times. Three positions during the theme were chosen for the analysis where the players inhaled the most (Figure 1): during the first part (A), between the two parts (B), and during the second part (C). All players inhaled at the transition between the two parts of the theme (B) and in the middle of the second part (C2). The mean movement trajectories in the knees and the arms across all players are shown in Figure 1.

At the melodic transition between the two parts of the theme (B), the players adopted an upright body posture with a mean knee value of 1.031 (SD = 0.035), which was significantly above 1 (*t*(19) = 3.96; *p* < 0.001). Additionally, they held their arms at a slightly higher angle than the mean angle with a value of 1.017 (SD = 0.035), again with significant difference to 1 (*t*(19) = 2.16; *p* = 0.022).

At point C2 (Figure 1), an eighth rest is noted in the score. At that point, all players adopted an upright body posture with a mean knee value of 1.024 (SD = 0.039), which was significantly above 1 (*t*(19) = 2.71; *p* = 0.007). The arms were at this point in a rather neutral angle with a value of 1.01 (SD = 0.037), without significant difference to 1.

**Figure 2:**
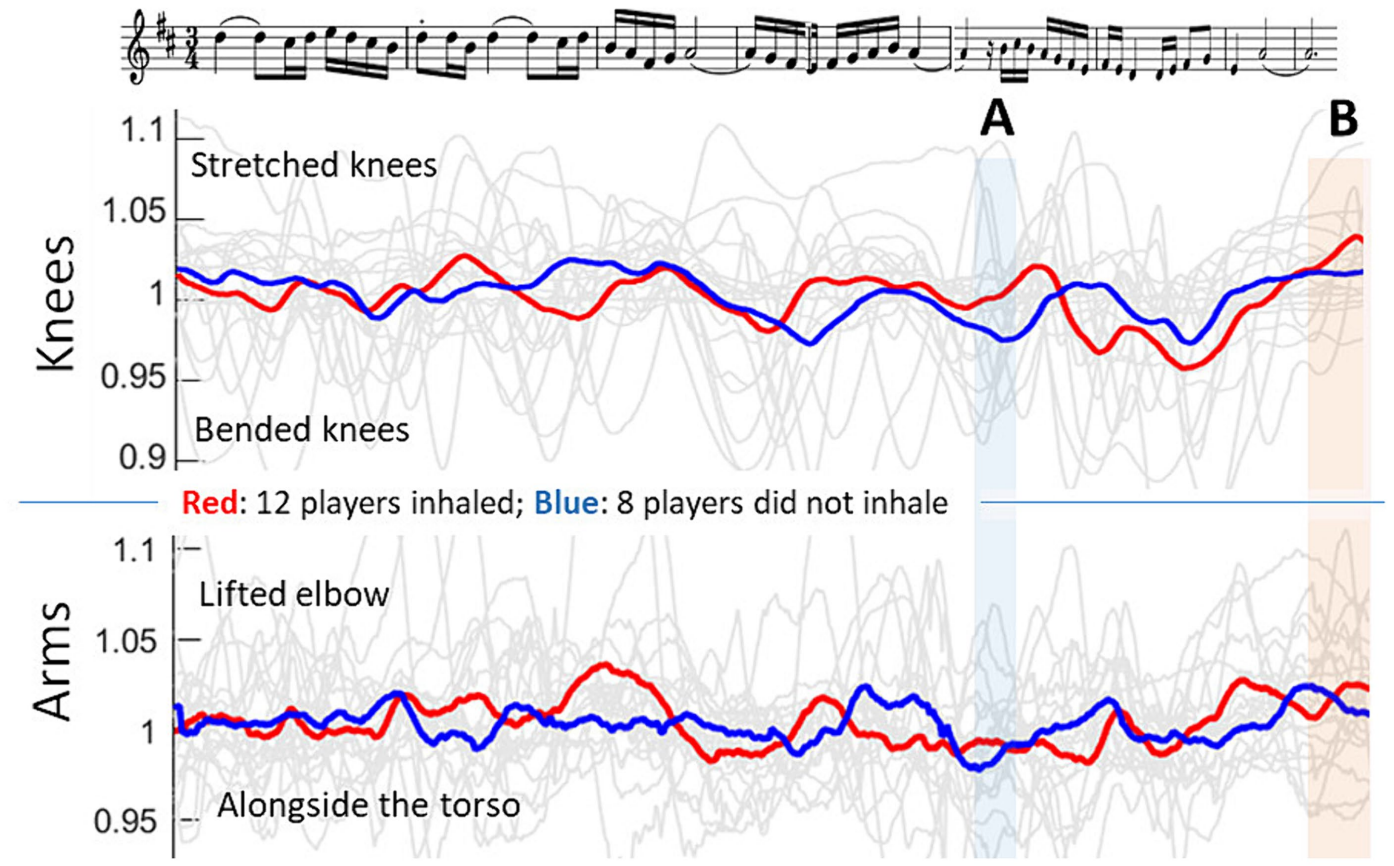
Top row: Score of Ravel’s “*Boléro*” theme created with LilyPond. Second and third rows: Trajectories of the angular movement in the knees and arms (in red: players who inhaled at point A; in blue: players who did not inhale at point A). A and marked with light blue: points in the theme where some players inhaled. B and marked in light orange: the middle of the theme. Please note that the score is slightly offset from the movement curves due to individual tempo variations and different lengths between written and played notes. Therefore, the score has been compressed or stretched at some points in order to adapt it to the timeline of the movement.

During the first part, 12 players inhaled at point A (Figure 1), where the 16th rest is noted in the score. The other eight players did not inhale at that point and played up to point B with one breath. The mean movement trajectories of the players who inhaled and who did not inhale were calculated separately. They are shown in Figure 2.

The mean knee angle of the players who inhaled at point A was 1.02 (SD = 0.021), which was significantly higher than that of the players who did not inhale (knee angle: 0.98; SD = 0.016; *F*(1,18) = 12.97, *p* = 0.002). The arm movement angle did not differ between those players and was rather neutral (arm angle: 0.99; SD = 0.027).

In the second part, the breathing involved more individual inhalations. While all players inhaled at point C2 (Figure 1), there were some spontaneous inhalations at points C1 and C3. In the area of C1, there have been very individual inhalations performed by seven players. The movement values of these players were taken at their inhalation moment and compared to a mean value around the same point in the piece of the other players. There was a significant difference in the knee angle values between the players who inhaled (knee angle: 1.01; SD = 0.031) and the players who did not inhale (knee angle: 0.97; SD = 0.029; *F*(1,18) = 10.5; *p* = 0.005). The arm movements were not significantly different between both groups of players (mean arm angle: 0.99; SD = 0.034).

At point C3, there was a short pause in the score where five players inhaled. However, all players performed on average at this point with a more upright body (mean knee angle: 1.022; SD = 0.037) and neural arm position (mean arm angle: 1.01; SD = 0.028). No significant difference was found between the players who inhaled and the players who did not inhale.

## 4 Discussion

This study investigated whether the movement behaviors of clarinetists followed different patterns when they inhaled during a performance compared to when the players did not inhale. The aim was to find out whether the performance changed as a result of the inhalation process and how this relates to expressive movements. Previous studies showed that when inhaling, clarinet players had a rather average body posture and their arms were at an average position (Weiss et al., 2018; Nusseck et al., 2022). The trajectories of the knee angles indicated that the players were at a rather upright body position shortly before inhaling and performed a knee bending right after inhalation.

The results showed that the clarinetists played with a variety of arm and knee movements that were mainly related to ancillary movement characteristics. Despite their very individual movements, at certain points in the theme, the players showed similar movement behaviors. At the transition between both parts (B), the body was brought into an upright position and the arms were put closer to the body. Here, the first part of the melody ended and all players inhaled. The movement behavior is therefore connected to an expressive intention, but also with the purpose of preparing for inhaling. Even the arms were put in a neutral position to the torso. This confirms previous findings that players performed at those transitions with a rather neutral posture even in a different musical piece (Nusseck et al., 2022).

A similar movement behavior was found in the middle of the second part of the theme (C2); at that point, again all players inhaled. The trajectory of the knees indicated that the players had an upright posture at this point. In addition, the arms were at a rather neutral position. This movement behavior, even during a melody, suggests the simultaneous use of both ancillary and instrumental movements, as the pause in the melody was expressively facilitated and the inhalation had to be prepared at the same time.

More interesting is the movement behavior at point A (Figure 2); at this point, not every player inhaled. The players who inhaled performed at that point with a rather upright posture. The knee trajectory even indicated that they seemed to prepare the inhalation with an anticipated knee stretching. In comparison, the players who did not inhale showed less supportive knee movement behavior and continued to perform with bent knees. After that point, they performed a brief knee stretch, which seemed to follow the normal knee movement pattern previously seen during their performance. The players who inhaled performed the second part of the first melodic phrase with even more knee bending than before. This finding indicates that the players who inhaled prepared for their inhaling by extending their legs, thereby providing a suitable posture for rapid inhaling. After the inhalation, they performed the remaining phrase with even more knee movements. This movement behavior differed from the players who did not inhale, which seemed to be ancillary movements that were more individual.

At another point in the piece (Position C1), again only some players inhaled. The exact point of the inhalation, however, was very individual. In the score, fast notes are to be played, whereby a very quick inhalation is possible between some notes. Here, the players who inhaled showed again a rather neutral and upright body posture, indicating preparation for the inhalation. The players who did not inhale performed around this point with uninfluenced, even movements. This finding clearly indicates a physiological function of the body movement to perform and prepare for the inhalation. The players briefly paused their musical expressive behavior to perform a physiologically oriented movement considering the respiration process and then continued immediately afterward with their individual expressive behavior.

The findings at point C3 in the second phrase support the facilitating and expressive purpose of the knee behavior. At this point, all players showed a similar behavior by adopting a more upright posture, regardless of whether they inhaled or not. It seems that this movement expresses the musical structure and was performed by each player. The players who inhaled at this point may have used this movement pattern to perform a quick inhalation.

### 4.1 Limitations

The study conducted was explorative in nature, aiming to investigate movement differences at specific points in a performance. Given the individual performances of the players, it is advisable to acquire more data at these points from a larger group of players. Furthermore, the individual reasons why some players inhaled at certain points while others did not need to be examined and taken into account. It could also be interesting to examine how the same player moves when inhaling and when continuing to play at the same point in a piece. To conduct such an analysis, a larger database needs to be compiled.

## 5 Conclusion

The findings showed that, in the course of the piece, there were several interplays of ancillary and instrumental movements. At certain points in the piece, the movement behavior of clarinetists were quite similar, indicating expressive functions in connection with the musical and expressive structure and showing physiological necessities to prepare for the inhalation. During the melody, the movement behavior of the players who had the urge to inhale was different from that of the players who continued to play without inhaling. This indicates the clear adaptation of a biomechanical movement to prepare for a physiologically supportive inhalation within the expressive movement behavior. On the other hand, similar movement behaviors of the players were found at a specific point in the melody that could also be used to perform a rapid inhalation. Despite being integrated into the performance, the individual movement and inhalation behavior were not explicitly exposed. Expert musicians can easily perform with ancillary movements that also constitute supportive elements for technical execution.

## Data availability statement

The raw data supporting the conclusions of this article will be made available by the authors, without undue reservation.

## Ethics statement

The studies involving humans were approved by Ethics commitee of the University Clinic Freiburg. The studies were conducted in accordance with the local legislation and institutional requirements. The participants provided their written informed consent to participate in this study.

## Author contributions

MN: Conceptualization, Data curation, Methodology, Project administration, Writing – original draft, Writing – review & editing. AI: Conceptualization, Writing – review & editing. JH: Formal analysis, Methodology, Writing – review & editing. CS: Conceptualization, Project administration, Supervision, Writing – original draft, Writing – review & editing.

## Footnotes

^1^ https://lilypond.org/
